# Supplementary material for: Evolution in caesarean section practices in North Kivu: Impact of caregiver training
Source: PLoS One. 2022 May 26;17(5):e0264251. doi: 10.1371/journal.pone.0264251 (PMC9135218; doi:10.1371/journal.pone.0264251)
Supplement: Attachment — Submitted filename: Response to reviewers.docx [file pone.0264251.s003.docx]

Dear Editor and the Reviewers,

We appreciated the careful revision of our manuscript, entitled ‘‘EVOLUTION IN CAESAREAN SECTION PRATICES IN NORTH-KIVU; IMPACT OF CAREGIVER TRAINING”. We would like to submit a revised version of the manuscript to the Journal. Your comments have been addressed point by point in the following way, and all the changes including main texts, tables and figures werehighlighted in yellow.

**Response to Academic Editor**

1. *It appears the authors must use multiple regression (only one dependent variable in each model) instead of multivariate (more than two dependent variables)*

Indeed, we have corrected in the methodology part and the text

2. *Please report regression prerequisite in method section and its handling methods.*

Indeed,we have just pointed out in the method section the prerequisites for multiple regression in order to make tables 1 to 4 understandable.

3. *Provide B: Unstandardized coefficients, S.E.: Standard error and Beta: Standardized* regression coefficients for variable and model Goodness of Fit in regression table.

Thank you for the remark, we have presented the OR(IC 95%) and the goodness of fit coefficient has been added.

4. Please present abbreviation (OR, AOR, ….) in their full format first time.

Thanks. Have been done.

5. *It appears in regression analysis reported in tables’ 5-7 only women with selected condition (only women’s with low birth weight, only women’s with neonatal mortality and only women’s with postoperative complications) considered as cases and were included in modeling and other cases were excluded.*

No, the first column only presented the percentages of the event, but the entire cohort has been analysed. This column has been removed from the tables for clarity.

6. *In regression analysis your dependent variable is women’s condition in relation to selected variables and group (Intervention – and Intervention -+) is your independent variable. And other factors were confounding factors.*

*Statistical analysis must be reviewed and corrected before more consideration.*

We completely agree with your comments .This brings us back to question number 2 concerning the consistency between the variables presented in tables 1 to 4 which were selected for the analyzes.

7. *In intervention section you declared “Following these findings, specific training was given to the local health staff.”, but we do not have any findings before this section.*

Thank you for pointing this. You are absolutely right; we have added these observations in the manuscript**.**

8. *Provide your ethic statement in method section and ethic committee approval form as appendix.*

The province of North-Kivu in the Democratic Republic of the Congo does not have an ethics committee, nevertheless the authorization to carry out this research was approved by the provincial health division in collaboration with the hospitals where this research has been carried out. We append, the authorization of the provincial authority

9. *In addition, the reviewers request improvements to the clarity of the written language.*

Thanks for the suggestion; English language editing has been improved through the manuscript

**Response to Reviewer #1**

*The objective of this study was to determine the impact of health staff training in a region of the DRC and which focused on the quality of C-section procedures by comparing outcomes determined at 2 sequential time points.
Multivariate logistic regression is used to determine which characteristics were associated with maternal and perinatal mortality and morbidity.
It is unlikely that dystocia or true fetal distress rates would be directly related to training, rather changes may be a reflection on how these diagnoses were defined and reported. A reduction in neonatal deaths is a “hard” indicator and maybe associated with the quality of training provided. The increase in scarred uteri may simply reflect a relative increase in the total number of prior cesarean sections. While training is relevant to ensure safe surgery practices for this group of women, in order to maximize study findings, it may be best to focus on outcomes among nulliparous women. Low birth weight is associated with a number of direct and indirect causes including access to adequate nutrition and the presence of micronutrient deficiencies, especially that of iron and folic acid.
Validated training and implementation of training procedures will likely not be a direct cause of untoward clinical outcomes. However, fetal distress is often cited as the reason for surgical intervention.
Cesarean sections performed in sub-Saharan African countries, while often indicated, are associated with higher rates of poor outcomes than those reported from other regions. The 3 Delays, including time to reach a facility as well as training and facility infrastructure must be addressed in future studies*

*Comments: We thank you for your very valuable comments*

*A few questions:*

1. *Was there a reduction in fistulas or reports of incontinence?*

No, we did not find a reduction in fistulas or reporting of urinary incontinence in our study. This could be due to the short scale of our study and also the fact that there was a lot of missing data for the retrospective study.

2. *How often was the WHO partogram used?*

We did not find any data on the useof partogram during our retrospective study. We will integrate this data in our next study

3. *Was there a difference in outcome between non-emergent c-section patients as compared to the two thirds of women who were considered emergencies?*

Yes, we observed a difference in outcome between the emergency cesarean section and the scheduled cesarean section,unfortunately we did not analyze statistically. Confers table 7

4. *During the second phase of this study, was more electronic monitoring equipment available?*Yes, if we make the comparison with that in the period before the training.
5. *Was there a report by the ERASMUS training team on their observations relative to the overall quality of care and challenges that need to be addressed?*

Yes, however there were many challenges to be met, including poverty, patient accessibility to healthcare structures, education and other socio-political and economic issues

6. *There has been a paucity of papers related to cesarean section rates and outcomes in the DRC. While it is difficult to draw meaningful conclusions from the data presented, the paper is still an important contribution in demonstrating the difficulties of addressing composite training needs in the face of limited resources and a challenging topography.*

Thanks. Agreed fully with your comments.There is indeed a dearth of articles on caesarean section in the democratic republic of the Congo, our team will continue to conduct research on this topic in order to contribute to reliable evidence.

**Response to Reviewer #2**

*Univariate and multivariate logistic regression was performed to determine which characteristics are associated with maternal and perinatal morbidity and mortality. These research methods are scientific and reasonable.

However, there are still some problems in this paper:*

1. *Baseline population was recruited from referral hospitals in the city of Goma from the period 01/11/2013 to 01/01/2016 (group 1).The second group was recruited from the same hospitals in Goma for the period from June 2019 to January 2020 (group 2). The time period of the two groups of people is not consistent, which may have an impact on the comparison results. It is suggested to explain this in the discussion or in the deficiencies of this paper.*

*Thanks for your comments. It is true that the time period in the 2 groups was not consistent and may have an impact on the comparative results. .We are aware and will take into account to avid bias. This comment has been added in the limitation of this study.*

2. *The writing of the article should be standardized. For example, all tables should be three-line tables. The article lacks key words. Thank you for the remark, the keywords are added in the manuscript and the tables were standardized.*

**Response to Reviewer #3**

1. *In the section of result，authors concluded that “Our study shows more cases of low birth weight, more post-operative complications, more maternal deaths, lower perinatal mortality… in group 2(after intervention) than in group 1(before intervention).”(as shown in Table 4). However, multivariate logistic regression did not support the conclusion. As shown in Table 5, intervention had no statistically significant impact on low birth weight (OR=1.9, P=0.13). As shown in Table 6, intervention had no statistically significant impact on neonatal mortality (OR=0.69, P=0.21).*

*Thanks for your comments. The statistic significant were not funded for those variables in the multivariable analysis which might be due to the small sample size. We adapted our sentence.*

2. *Given the fact that there are significant differences in socio-demographic characteristics, medical data and obstetric characteristics between the two groups, multivariate logistic regression result is more reliable. Also, the result of multivariate logistic regression should be added in the abstract.*

*Thanks for your useful comments. The intervention variable is added in table 7 and the result of the multiple regressions is added in the abstract. Multiple logistic regression would be more reliable for socio-demographic characteristics, medical and obstetric data.*

3. *Is it reasonable to choose low birth weight, maternal deaths, and perinatal mortality as the evaluable indicator? The objective of the study is to assess the impact of health staff training on the quality of CS procedures. Firstly, low birth weight cannot reflect the quality of CS procedures very well; Secondly, the incidence rate of maternal deaths, and perinatal mortality is very low, it is hard to measure a significant difference (unless the sample size is very large). Maybe the indicator such as operative time, blood loss, APGAR, duration of stay and post-operative complications are more suitable.*

*Thanks for your valuable comments. We used low birth weight , maternal and perinatal death as indicators. However, your remark on low birth weight rate of maternal and perinatal mortality is low in our study.We will take into account during our large-scale study to analyze indicators such as operating time,blood loss,APGAR,duration of stay and post operative complications.*

4. *APGAR and duration of stay were compared between the two groups in the study, but the result did not described in the abstract.*

*Thanks for pointing this.Due to limited words in the abstract, we just presented main results.*

5. *Although there is no statistical significance in APGAR between the two groups by Chi² Pearson test, a multivariate logistic regression should be added to assess the impact of intervention on APGAR.*

*Thanks. Following your suggestion, this has been done, the results were similar. We indicated the results as the text in the manuscript.*

6. *In table 7, the variable “intervention” should be added. Also, multivariate logistic regression result should be added to assess the impact of intervention on post-operative complications.*

*Thanks. Done.We could not do multivariable analysis because the number of cases was small (n:16)*

7. *In the study, statistical analysis is performed appropriately, but the interpretation of the statistical results requires improvement. In the abstract, “Our study shows a decrease in neonatal deaths, dystocia and foetal distress, but an increase in post-operative complications, maternal deaths and cases of scarred uterus and low birth weight.” Among them, dystocia, foetaldistress and scarred uterus is the obstetric characteristics of maternal, which can hardly be influenced by health staff training and cannot reflect the quality of CS procedures very well*

*Thanks. We agree with you that the training can help health care staff to correctly ask indications for cesarean section such as dystocia, fetal distress, scarred uterus but may not properly reflect the quality of the cesarean section.*

8. *Some data should be checked. For example, the data of post-operative complications in table 4 should be checked.*

*Thanks, Verification and corrections have been done. In the abstract “In the population studied prospectively, after the implementation of health staff training, there were fewer cases of dystocia, foetal distress and neonatal death, but more cases of scarred uterus, post-operative complications and low birth weight.” The word “cases” is not used properly. May be “incidence rate” is more suitable.*

*Thanks. Corrections have been done.*
