# Supplementary material for: Evolution in caesarean section practices in North Kivu: Impact of caregiver training
Source: PLoS One. 2022 May 26;17(5):e0264251. doi: 10.1371/journal.pone.0264251 (PMC9135218; doi:10.1371/journal.pone.0264251)
Supplement: Attachment — Submitted filename: Response to Reviewer Comments October 26Nov-WHZ.docx [file pone.0264251.s004.docx]

Dear Editor,

We appreciated very much the valuable comments of the reviewersthat helped us to improve the quality of the paper. Hereby we submit a revised version of the manuscript. Below, we have included the point-to-point responses to the reviewers’ comments. All the changes including main texts, tables and figures were highlighted in yellow.

**Comments of Reviewer #1**

**General comments**: Globally, cesarean section continues to remain on the rise, and in some cases have led to improvement in maternal and neonatal outcomes. Unfortunately, such is not the case in much of sub-Saharan Africa, not obvious from the data reported in this paper where there was no neonatal benefit and 1 in 500 women died perhaps as a result of this procedure. Undoubtedly, the death rates were even greater, as few women present for a postpartum visits, yet it is in this period that many cases of postpartum sepsis and its sequelae are to be found.A comparison of complications among two groups of women based upon the year they delivered was well described. These were a heterogeneous grouping with significant differences in baseline characteristics.

Poor outcomes linked to cesarean section births are multifactorial and the authors correctly point out areas that are not easily addressable without a major infusion of resources.

**Response:**Fully agreed with your comments and thank yourencourage words!

**Comment 1**. The data presented is primarily from a tertiary care (transfer) facility. Given the difficult geography of the DRC, it is not surprising that by the time a woman reaches a tertiary care hospital, many of the cesarean sections are characterized as urgent – a designation associated with much poorer outcomes.

**Response:** Thank you for your valuable comment which we agree.

**Comment 2.** Rates of cesarean section in many parts of sub-Saharan Africa have remained at 4-5%. With 17-19% cesarean section rates cited in this paper from a referral facility, is important to report on the details associated with patient access, delays, and referral

**Response:**It is true that cesarean section rates in most parts of sub-Saharan Africa have remained very low compared to the WHO recommended rate which is around 10 to 15%.The rate of 17 to 19% described in our study is due to the conditions of transfer to referral hospitals, the accessibility of these hospitals, the level of education of the population in relation to prenatal consultations, the training of medical personnel.As these hospitals are referral centers where they receive more transferred cases and preformed more emergency cesarean sections.

**Comment 3. I**t is counterintuitive to believe that skills training would not accrue positive benefit, but the author correctly point out that the diagnosis of dystocia or fetal distress are often unclear and may lead to unnecessary operative deliveries.

**Response:**Thanks for your comments which we agreed that the diagnosis of dystocia or fetal distress are often unclear and may lead to unnecessary operative deliveries.

**Comment 4**. Socio-economic status, distance from a hospital and whether a woman obtained prenatal care has been shown to be important in predicting risk.

**Response:**We agree with you that socioeconomic status, distance to hospital and prenatal consultations are very important in predicting the risk of cesarean section and maternal and perinatal complications.

**Comment 5**: From the data presented, it is difficult to prioritize future training needs. However, the importance of assuring generalizability among sites and reporting on antenatal risk factors would be helpful in analyzing future outcome data.

**Response:** Thanks for pointing this which could be included in the future data analysis.

**Comments of Reviewer #2**

**Comment 1.** The author has modified most of the questions according to my revision opinions, except for a few minor questions, namely:Table5-table7 were not standardized

**Response:**Many thanks for your kind reminder which revision has been done by standardizing the tables 5-7.

**Comments of Reviewer #3**

**Comment 1.** Please add the training time to the “Intervention” section, including when to start the training and the duration of the training

**Response:**Thank you for your valuable comments. We have revised the intervention section by providing the information about the starting time and duration of the training.

**Comment 2.**There is still room for improvement in the description and interpretation of the results. For example, In “Abstract” section “after the implementation of health staff training, there were fewer incidence rate of dystocia, foetal distress and neonatal death, but more incidence rate of scarred uterus, post-operative complications and low birth weight.”

It is not appropriate to use obstetric characteristics of maternal (dystocia, foetal distress, scarred uterus) to evaluate the effect of intervention. This description makes readers feel that training is the reason for the increase of scarred uterus. It is more appropriate to evaluate the training effect with outcome indicators, however, low birth weight is not recommended as an evaluation indicator of intervention effect. It is meaningful to explore the influencing factors of low birth weight, but the purpose of this paper is to evaluate the effect of training.

**Response:** Thanks for your valuable comments**.** The description and interpretation of the results have been improved in the summary section..We agree with you that the main aim of this study is to assess the impact of training of medical personnel on the improvement of the cesarean section.We will take into account in our next study not to use the obstetric characteristics of the mother and the low birth weight to evaluate the impact of the intervention.

**Comment 3.**In the paper, “Total duration of stay in days”， “patient’s file is complete”“blood transfusions performed” are reported, and there are statistical significances between the two groups. These indicators are appropriate to assess the effect of training. So it is suggested to describe the relevant results in the abstract.

**Response: Thanks for useful comments.** We revised this in the result part.

**Comment 4.**Although the results of data analysis do not suggest that training is effective in reducing neonatal death and post-operative complications, the research is meaningful. Analyzing the reasons behind the result and putting forward targeted countermeasures for risk factors of neonatal death and post-operative complications will increase the practical significance of this study.

**Response:** Thanks for your valuable comments with which we fully agreed.
